# Skin scarring: Latest update on objective assessment and optimal management

**DOI:** 10.3389/fmed.2022.942756

**Published:** 2022-10-05

**Authors:** Rubinder Basson, Ardeshir Bayat

**Affiliations:** ^1^Wound Healing Theme, NIHR Manchester Biomedical Research Centre, Centre for Dermatology Research, University of Manchester, Manchester, United Kingdom; ^2^Wound Healing Unit, Medical Research Council (South Africa), Division of Dermatology, University of Cape Town, Cape Town, South Africa

**Keywords:** scar, skin, topical - skin cream, keloid, hypertrophic, randomized clincial trial

## Abstract

Although skin scarring is considered by some to be a minor, unavoidable consequence in response to skin injury, for many patients, cosmetically unsightly scars may cause uncomfortable symptoms and loss of function plus significant psycho-social distress. Despite their high prevalence and commonality, defining skin scars and their optimal management has proven problematic. Therefore, a literature search to assess the current evidence-base for scarring treatment options was conducted, and only those deemed Levels of Evidence 1 or 2 were included. Understanding the spectrum of skin scarring in the first instance is imperative, and is mainly comprised of four distinct endotypes; Stretched (flat), Contracted, Atrophic, and Raised for which the acronym S.C.A.R. may be used. Traditionally, scar assessment and response to therapy has employed the use of subjective scar scales, although these are now being superseded by non-invasive, objective and quantitative measurement devices. Treatment options will vary depending on the specific scar endotype, but fall under one of 3 main categories: (1) Leave alone, (2) Non-invasive, (3) Invasive management. Non-invasive (mostly topical) management of skin scarring remains the most accessible, as many formulations are over-the-counter, and include silicone-based, onion extract-based, and green tea-based, however out of the 52 studies identified, only 28 had statistically significant positive outcomes. Invasive treatment options includes intralesional injections with steroids, 5-FU, PDT, and laser with surgical scar excision as a last resort especially in keloid scar management unless combined with an appropriate adjuvant therapy. In summary, scar management is a rapidly changing field with an unmet need to date for a structured and validated approach.

## Introduction

To many, a scar is often considered trivial, an accepted outcome as a result of an operation or trauma; but to some, a scar which does not fade to an acceptable degree can be disfiguring causing a considerable psychosocial impact on that individual (1), not to mention discomfort, itching, or even pain and tenderness, and in some cases where physical contractures may form, scars can result in disability or loss of function. The degree of scarring depends upon many factors including the individual’s own genetically inherent healing capabilities, site and extent of injury (2). Regardless of clinical specialism, all clinicians will encounter skin scars during their career; this article provides an evidenced-based update on objective assessment and optimal scar management.

## Methods

A literature search conducted in PubMed identified clinical trials to assess management options to treat skin scars. Search terms included*: skin scarring AND (treatment OR management) AND (clinical OR randomized trial).* To isolate the highest quality studies, the Oxford Level of Evidence protocol was employed so that only Levels 1 and 2 (systematic/meta-analyses of randomized controlled trials, (RCTs)/high quality RCTs; and systematic reviews of cohort studies and low quality RCTs) were included (3). Only scars formed post-acute wounds, traumatic skin injury or surgery excluding chronic wounds were included, therefore chronic wound management studies were totally excluded. Studies regarding surgical excision, skin grafts, skin flaps and suture techniques were also excluded as these techniques can be considered as a separate entity. In addition, extensive scientific and clinical experiences (spanning over 20 years) in management of skin scarring from the senior author is herein shared.

## The clinical burden and cost of scarring

In excess of 20 million people are affected by skin scarring in the United Kingdom alone, of which nearly a quarter, claim that it has caused short term emotional and/or physical problems (4). Fourteen percent claim long term physical or psychosocial disability (4). The burden of clinical management of skin scarring costs the NHS £8.3 billion per annum (5). The most common clinically managed form of scars in children occur as a result of burns injuries; (6) in 2019 alone, 64 000 children sought medical attention for burns (6). Pediatric scalds from just hot baths cost the NHS £39.2 million, yet this is an area of medicine that is poorly funded and understood, and therefore treatment strategies are often inadequate or ineffective (6). It is even more difficult to estimate the number of people affected by scarring worldwide: in the developed world alone, over 4 million people are affected by symptomatic scarring requiring management each year (7); however these figures only reflect scars caused by trauma (e.g., burns), and do not take into account the number of scars created by emergency and elective operations which will heal with varying degrees of success. Regardless of the cause, a third of all patients will suffer pathophysiological aberrations such as hypertrophic and keloid scars (8), making them even more likely to seek treatment to improve symptoms and scar cosmesis.

## Types of scars

**FIGURE 1:**
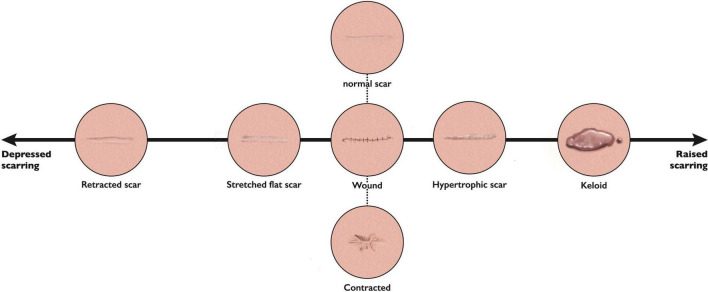
The spectrum of scar endotypes. This figure represents the scar phenotypes discussed, with retracted or depressed (atrophic) scars on one side, stretched (flat) scars closer to normal skin in appearance, through to raised phenotypes (hypertrophic and keloid) at the other end in terms of the range of scar phenotypes/endotypes.

A spectrum of skin scarring exists however due to their significant variability and heterogeneity, classification of scars has often proved troublesome, with a distinct lack of objective, standardized methods (9). With a recent move toward precision medicine (10), and therefore moving away from the “one size fits all” approach which is often adopted in skin scar management, a better understanding of phenotypes and endotypes is required. This phenomenon has already been adopted in allergic diseases, such as asthma, and atopic dermatitis (10). In the context of scarring, phenotypes, (or visible properties which may be observed), include redness or scar thickness for example, whereas an endotype can be defined as the pathophysiological mechanism underlying said phenotypes (11). Clinicians may observe elevation or depression of scar tissue, and altered color or texture. Very often, scar formation is site dependent: raised when crossing Langer’s lines, contracted over joints, or stretched where a wound is placed under tension (2). Particularly challenging, are a variant of raised scars which extend beyond the margins of the original site of skin injury, known as keloid (12). They do not show signs of regression with time, and often continue to progress (12). The spectrum of abnormal skin scarring however, is mainly comprised of four distinct endotypes (11), (1) Stretched (flat), (2) Contracted, (3) Atrophic (depressed), and (4) Raised; for which the acronym S.C.A.R can be a useful aide memoire (Figure 1) (11). Phenotypes (scar features) may be present in more than one endotype. A stretched, (flat) scar, is the least symptomatic, and closest to a normal “fine line” scar (generally considered as a normal scar phenotype), although the latter will be closer to the patient’s normal skin color, and be symptomless (11). Stretched scars often occur when wound closure is under tension. Contractures, in contrast, most commonly occur following burn injuries, and their development is strongly influenced by closure under tension and site of injury; if adjacent to joint surfaces they may result in significant pain and reduced function for the patient (13). Atrophic (depressed) scars, develop below the surface of the surrounding skin, such as acne scars (14). Finally, the raised scar endotype, comprising hypertrophic and keloid scars, are considered the most significant in terms of cosmetic, symptomatic and psychological impact on the patient (2). Ethnicity plays an important part in keloid scarring, with prevalence higher in individuals with darker pigmented skin, namely those of African, Asian, and Hispanic descent (15). Keloid scarring may also be familial, indicating a genetic predisposition in some individuals, although interestingly, is unique to humans; as no animal model exists (15).

## Scar assessment

**FIGURE 2:**
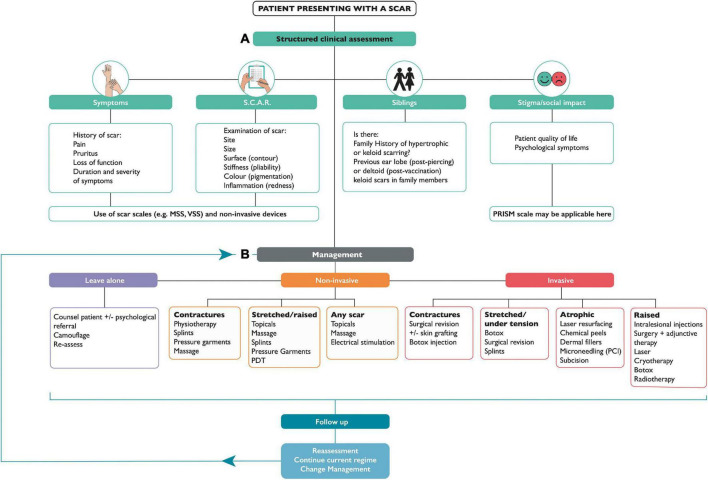
Flow chart for assessment and management of skin scarring. Part **(A)** Discusses structured clinical assessment. **(B)** Represents management options for skin scars, which may follow one of 3 options: leave alone, non-invasive, or invasive treatment. NB. S.C.A.R -(Stretched, Contracted, Atrophic, Raised); MSS- Manchester Scar Scale; VSS- Vancouver Scar Scale; PRISM- Patient Reported Impact of Scars Measure; TAC- Triamcinolone; 5-FU- 5-FluoroUracil; PDT- Photodynamic Therapy.

**FIGURE 3:**
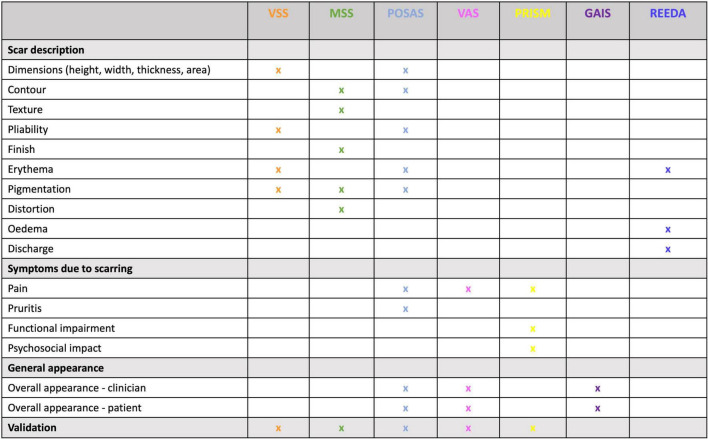
Scar scale summary. This table allows for the comparison between scales used for scar assessment according to their appearance, signs and symptoms, and, whether they include patient or clinical perspective. VSS – Vancouver Scar Scale; MSS – Manchester Scar Scale; POSAS- Patient and Observer Scar Assessment Scale; VAS- Visual Analog Scale; PRISM- Patient-Reported Impact of Scars Measure; GAIS- Global Aesthetic Improvement Scale; REEDA- redness, edema, ecchymosis, discharge, approximation.

Patients presenting to a clinician with a scar complaint, whether in a primary or secondary care setting, will often have unrealistic expectations; therefore, understanding and managing these expectations is just as equally important as an accurate diagnosis and an appropriate management plan. A structured scar assessment is crucial (Figure 2): clinicians should take an appropriate history (including family history), and examine the patient, including photographs before and after treatment. During history and examination, it is helpful to remember key components of a scar such as the 4 “S’s”: (1); S.C.A.R (endotypic features, as described above), (2); Symptoms (may include itching and pain or loss of function for example), (3); Siblings (is there a positive family history?) (4); Stigma or Social implications (how is the patient’s quality of life affected, psycho-social as well as physical well-being?). Scar scales are a common-place tool used to monitor the severity of a scar and its response to treatment, although they possess an inherent weakness due to their subjective nature and variability. Our review found scar scales were the most commonly employed method for assessment of therapeutic response, namely the Vancouver Scar Scale (VSS) initially intended only for burn scar evaluation (16), yet it has been frequently used to assess many scars regardless of etiology as has the Patient and Observer Scar Assessment Scale (POSAS). Both assess vascularity, pigmentation, and pliability, the VSS assesses height, whereas the POSAS uses surface area, the latter also takes into account the patients’ perspective of symptoms (9). In both cases a lower score indicates less severe scarring. The Patient-Reported Impact of Scars Measure (PRISM) scale, is the first scar-specific scale which uses the patient’s perspective (often misunderstood by clinicians), as an outcome measure, addressing both symptoms and quality of life (17); a lower score indicates less severe scarring. The Manchester Scar Scale has been used in clinical practice (1), and takes a slightly different approach using a visual analog scale, but again cannot truly account for the nature of all scar types as its intended use was for raised skin scars such as hypertrophic and keloid scars (1). Figure 3 summarizes all the scales that have been used to measure skin scarring.

More recently, non-invasive devices have been employed, providing objective, quantitative measurements on a variety of parameters (such as erythema and pigmentation) and can evaluate changes in comparison to baseline measures of the skin scar. In a recent study by Lee et al. (18) the intra- and inter-rater reliability of both subjective and objective measurements for analysis of burns scars, using mVSS and POSAS, patient satisfaction questionnaires and a range of devices including ultrasound, colorimeters and cutometers were analyzed. Their study found that the majority of parameters on scar scales when performed by less than 3 assessors had poor reliability, whereas the objective devices ranged from acceptable to excellent. Interestingly, there is still a role for subjective measurements, particularly from the patients’ perspective, as they rated symptomatic relief of pain and itch above appearance (scar thickness and color) (18). Lee et al. also reviewed objective devices for burn scar analysis classified according to the features they may assess (9). These included color, for example digital photographs and laser imaging; scar dimensions, using 3D photographic imaging and ultrasound; texture- skin topography, also measured using the aforementioned techniques; biomechanical properties such as pliability and elasticity, measured by cutometers, tissue tonometry, and elasticity (suction) probes; physiological disturbances, (hydration and water loss measured by various probes placed over the scar, or multispectral imaging systems); and non-invasive morphological imaging, where ultrasound, optical coherence tomography, microscopy, and spectroscopic techniques may be employed (9). This study demonstrates the vast array of devices that may be used to obtain objective measurements and highlights the many factors which may be used to assess scar severity and the desperate need for better classification of skin scars. However, it is important to note the high cost both to purchase and service these devices (9, 18, 19), and therefore accessibility to them, especially when in most cases, they were not designed principally for scar assessment. Despite this, their use can span all four phases of wound healing (19), and although there is not one single device which can measure all parameters simultaneously, many offer multi-probe systems (19) which can validate observed positive findings in the clinical setting, when monitoring treatment response.

The difficulty however, is that non-invasive devices are still mostly used for research purposes alone and have not become mainstream in clinical practice.

## Current management strategies

**FIGURE 4:**
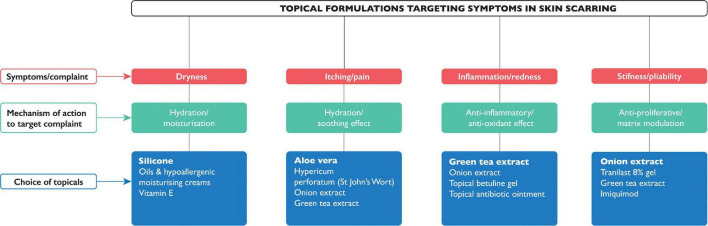
Known topical formulations for managing symptoms in skin scarring. The symptoms (in red) that topical formulations claim to target, with their mechanism of action (in green), and commonly available topicals (in blue); the topical most cited or with the highest evidence is highlighted in bold.

When it comes to management for skin scarring, treatment options are varied, depending on the nature and type of the scar. Therapeutic options include adopting a leave alone strategy, use of non-invasive, or invasive approaches. Awareness of when not to intervene is key, and a ‘‘watch and wait’’ approach is clinically appropriate in certain cases, particularly of young scars in cosmetically susceptible anatomical locations; where frequent review may elicit a reduction in symptoms, improvement in cosmesis, or improved psyche of the patient who has come to terms with living with the scar and where invasive intervention may lead to potential undesirable adverse side effects (e.g., depigmentation and lipoatrophy post-steroid injection). In the short term, however, exploring patient anxieties and also cosmetic camouflage can equally prove useful as an adjunct to increasing self-confidence while waiting for skin pigmentation to settle. The British Association of Skin Camouflage is a useful resource.^1^ There are also many options encompassed under non-invasive alternatives. If scars are present across joints, it is important to maintain movement through exercises (*via* physiotherapy and occupational therapy referral) to minimize scar contractures. Other aids such as splints, and pressure garments, may be used prophylactically to minimize potential stretched or raised scar formation. Offloading tension through the application of pressure on a wound (20) will create better outcomes in terms of cosmesis; therefore, pressure garments have been widely used although with varying degrees of success dependent on the nature of the treatment protocols and quality of the garments. However, poor patient compliance remains the main issue as the patient is required to wear the garment daily for 23 h a day in order to see significant results (20, 21). Additionally, there are several topical formulations, many of which are available over-the counter, and therefore easily accessible without the need for medical consultation/referral and/or a doctor’s prescription. Of the 162 of studies identified in this review, over a third (*n* = 52) were for topical formulations (Figure 4), however only 28 had statistically significant positive outcomes (see Supplementary Table 1). Most outcome measures were assessed using scar scales, while a few also used non-invasive quantitative device measurements. Silicone-based topicals (22–42), which are a widely accepted treatment for symptomatic relief and improvement in scar cosmesis, are supported by few level 1 and 2 evidenced-based trials (38). Even tough silicone formulations which are inert and inherently lack an active, are presumed to be particularly effective for prevention of dryness, by providing occlusion and hydration which helps improve the skin’s overall condition. Natural-based topicals such as *Aloe vera* (43, 44), used to tackle itch, green tea (*Camellia sinesis)* (45) and onion extract (*Allium cepa*) (46–54), both of which have a potent anti-oxidant as well as anti-inflammatory effect, have been shown to reduce inflammation and prevent overproduction of collagen, in clinical trials (level 1 evidence) to support their use in the management of scarring. Of note, a recently published double blind randomized clinical trial provided evidence (level 1) of the role of a topical containing a potent green tea extract termed EGCG (Epigallocatechin gallate) known for its anti-oxidant and anti-inflammatory activities in significant reduction of skin scarring if applied pre-emptively pre-surgery (55).

It has even been proposed that it is not necessarily the topical, but the action of massage that has an effect on scar appearance. However, even in recent studies, whilst the effect of massage accelerated improvements in scar elasticity and thickness early on, there was no perceived long term benefit (56). There is also a role for electrical stimulation, which has been shown to suppresses collagen I formation in keloids, and alleviates symptoms such as pain and itching (15).

Several studies have demonstrated the efficacy of laser therapy in the treatment of raised scars. Although there are specific types of lasers depending on depth and pigment target including pulse-dye laser (PDL) (for erythema), carbon dioxide, (CO_2_) (for ablation), neodymium-doped yttrium aluminum garnet (Nd: YAG) (for post-inflammatory pigmentation). More recently, laser-assisted drug delivery (LAAD), is popularized where a topical agent is applied to a targeted area, as a “two-pronged” attack (57). This approach is thought to increase the bioavailability of the drugs used, and shown to be of benefit in keloids (57). Photodynamic therapy (PDT) is widely used to treat skin cancers due to its cytotoxic effect, induction of fibrotic tissue degradation, by decreasing collagen I and III gene expression is now also used for treatment of raised scar endotypes, although current evidence on therapeutic efficacy is limited (58). Injections with steroids, principally triamcinolone, (TAC), intralesional 5-fluorouracil (5-FU) (59) inhibiting proliferation, which act to reduce inflammation and cause ECM (extracellular matrix) degradation and reduce collagen synthesis, have been frequently used for managing keloid scars with variable outcome depending on the site and severity of the keloid scars being treated. Additionally, matrix metalloprotease-2 (MMP-2) induction (59), and interferon therapy (60) though currently not in common use, have been tried. Intralesional bleomycin and verapamil also reduce collagen synthesis, and verapamil also increases collagen degradation (60). For keloids however, the most cited treatment is excision, with and without adjuvant therapy (often with steroid injection or radiotherapy) despite high recurrence rates (61).

Injections with botulinum toxin A (BTA) can relax muscles where wounds are under tension and enhance the remodeling process (25). BTA has also been found to reduce collagen production and reduce hypertrophic scars (25). As regards to atrophic scars, chemical peels, laser resurfacing, and dermal fillers are the most commonly used options. Recently, microneedling, or percutaneous collagen induction (PCI) therapy has also been employed (62). The repetitive puncture of the dermis using microneedles is considered to trigger growth factors to stimulate collagen plus elastin production, as well as angiogenesis. A systematic review published earlier this year demonstrated an efficacious role for microneedling in managing atrophic acne scars (63).

## Future perspectives

**FIGURE 5:**
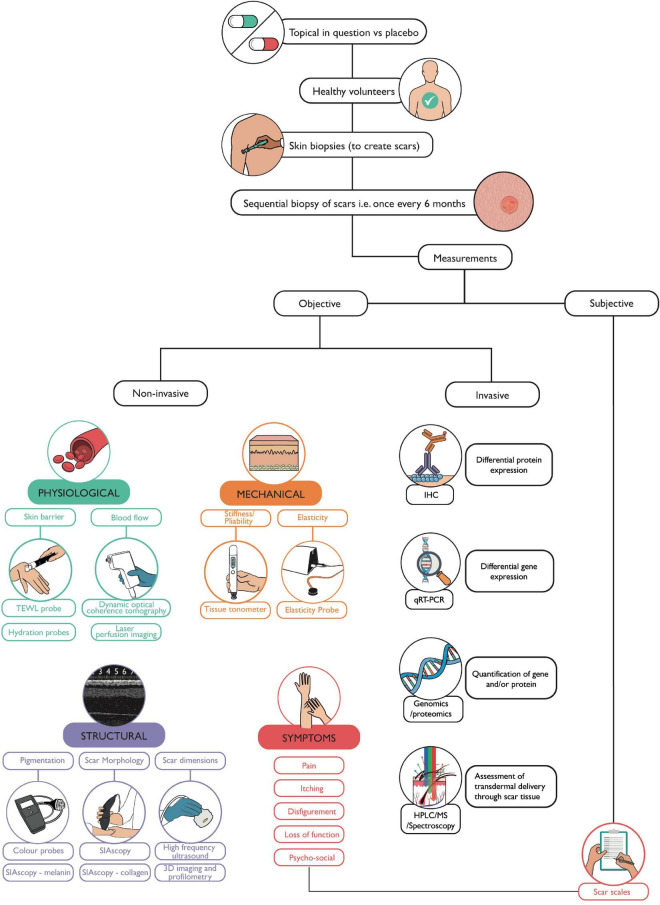
Workflow for functional evaluation of a topical. This flow diagram depicts the methodology we propose which can be used to assess any topical, in the absence of a current gold standard. Skin biopsies may be taken from healthy volunteers to create scars, for example, on both arms. The volunteer can then be randomly assigned treatment and placebo topicals to apply to scars on each arm. Sequential skin biopsies can then be taken of the scars to allow for in-depth laboratory analysis. Meanwhile, prior to each biopsy, non-invasive devices can be used to measure the physiological, mechanical, and structural parameters of a scar at each visit compared to the individual’s baseline (normal skin) measurements. Techniques such as H&E, IHC (immunohistochemistry), q-RT-PCR (quantitative reverse transcriptase polymerase chain reaction), and gene/protein/metabolite quantification can validate findings from non-invasive measurements. High performance liquid chromatography (HPLC), mass spectrometry, or spectroscopic techniques such as Raman spectroscopy, can be used to assess transdermal delivery and penetration of topicals through scar tissue. The volunteer’s perspective is also accounted for through the use of scar scales describing symptoms and the extent and impact of scarring on that individual.

With the advent of precision medicine, soon becoming the optimal choice in patient management, a diagnostic and a theranostic paradigm shift in scar therapy is about to take place. However, it is evident that further clinical and scientific translational research in skin scarring pathobiology is required to help identify bespoke biomarkers and devices aiding in diagnosis and helping in monitoring of response to treatment. The use of temporal, sequential biopsies to allow for invasive analysis, coupled with non-invasive skin scar measurements by using quantitative devices is validated by emerging studies (45, 64–67) (Figure 5). Understanding mechanisms of skin fibrosis, could help navigate toward a more desirable and currently unattainable goal of scar-free healing (scarless healing can occur in early gestation mammalian embryos) (2).

Reducing mechanical stress on wounds can play a role in reducing pro-inflammatory and fibrotic mechanisms during wound healing; polymer-based medical devices have been successful in reducing scarring in surgical incisions (68). Currently there are prototypes of medical devices which target molecular mechanisms involved in mechanotransduction, such as downregulation of focal adhesion kinase (FAK), of which overactivation can result in hypertrophic scar formation (68). Other devices include skin substitutes comprising human embryonic stem cells and induced pluripotent stem cells, however their use may be hindered by ethical concerns (68).

Focusing on mechanical stress has also led to the discovery of another mediator of wound healing, *Engrailed-1, (EN-1)* (69). When tension was applied to wounds in a mouse model, there was an increase in *EN-1* expression, resulting in a thicker wound (69). The application of a drug, verteporfin (principally used to treat age-related macular degeneration) to the same wounds, did not only reduce scarring, but regenerated the skin (complete with appendages such as hair follicles and sebaceous glands not present in scars) (69).

Recent research has also demonstrated that there may be a role for lung surfactant which reduces alveolar surface tension in preterm infants (70). Lung surfactant, comprising of phospholipid films with surfactant proteins, interferes with cell signaling blocking pro-inflammatory mediators and thus reducing inflammation (70). It was therefore hypothesized and demonstrated in a phase I clinical trial that application of lung surfactant on wounds would reduce the inflammatory process and accelerate healing, suggesting that there may be an innovative role for lung surfactant in the treatment of wounds in the future (70).

## Key messages

1. Over 25% of people with scars in the United Kingdom suffer short term psychological or physical problems as a result, yet skin scar management remains poor, with generic and costly strategies employed which do not take into account scar endotypes.
2. The spectrum of abnormal skin scarring is mainly comprised of 4 distinct endotypes, (1) Stretched (flat), (2) Contracted, (3) Atrophic (depressed), and (4) Raised, (the acronym S.C.A.R). Non-invasive devices can be used to define scars, and provide quantitative and objective analysis, which scar scales are lacking.
3. Current management strategies include (1) leave alone, (2) non-invasive, or (3) invasive treatment. Topical formulations, largely available over-the-counter, are popular, yet surprisingly the evidence for their efficacious claims is limited.
4. A better understanding of the pathogenic mechanisms of skin fibrosis is required to before we reach the currently unobtainable goal of scar-free healing.

## Author contributions

RB: construction, research, and writing. AB: conceptualization and design of the review article, extensive subject matter clinical and research oversight and expertise, editing, and proofreading. Both authors contributed to the article and approved the submitted version.

## Footnotes

1
https://skin-camouflage.net/scwp/

## Supplementary material

Supplementary Figure 1 Literature search flow chart and levels of evidence table. **(A)** Flow chart evidencing studies with inclusion and exclusion criteria and **(B)** Oxford Levels of Evidence (only Levels 1 and 2 included for review, as demonstrated in part a).

Supplementary Table 1 Topical formulations for skin scarring. An array of topicals exist for skin scar treatment, Table 1 highlights those ranking as Level 1 or Level 2 LOEs.
