# Supplementary figures and images for: Skin scarring: Latest update on objective assessment and optimal management
Source: Front Med (Lausanne). 2022 Oct 5;9:942756. doi: 10.3389/fmed.2022.942756 (PMC9580067; doi:10.3389/fmed.2022.942756)

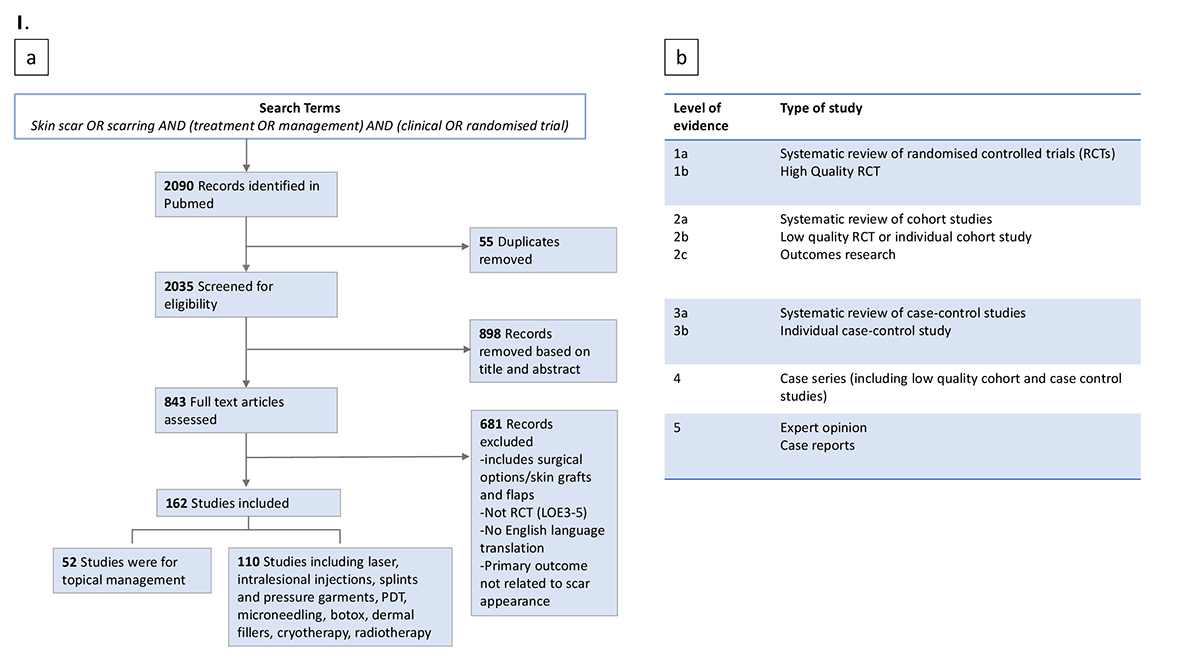

Supplement: Supplementary Figure 1 — Literature search flow chart and levels of evidence table. (A) Flow chart evidencing studies with inclusion and exclusion criteria and (B) Oxford Levels of Evidence (only Levels 1 and 2 included for review, as demonstrated in part a). [file Image_1.TIFF]
