# Supplementary material for: Skin scarring: Latest update on objective assessment and optimal management
Source: Front Med (Lausanne). 2022 Oct 5;9:942756. doi: 10.3389/fmed.2022.942756 (PMC9580067; doi:10.3389/fmed.2022.942756)
Supplement: Supplementary Table 1 — Topical formulations for skin scarring. An array of topicals exist for skin scar treatment, Table 1 highlights those ranking as Level 1 or Level 2 LOEs. [file Table_1.DOCX]

| **Topical** | **Authors** | **Number of cases** | **Outcome** |
| --- | --- | --- | --- |
| Silicone | Wiseman et al, 2020  Moortgat et al, 2019  Nor et al, 2017  deGeorgi et al, 2009  Cadet et al, 2018  Kong et al, 2014  van der Wal et al, 2010  Karagoz et al, 2009  Meseci et al, 2017  Jenwitheesuk et al, 2012  Song et al, 2018  Wananukul et al, 2013  Keorochana et al, 2015  Palmieri et a; 1995,  Surakunprapha et al, 2020  Kim et al, 2014  Kim et al, 2016  O’brien and Jones, 2013  Gold et al, 2001 | 153  60  17  110  12  100  23  45  39  60  60  30  70  80  46  30  33  873  96 | Negative/neutral  Positive  Negative/neutral  Positive  Negative/neutral  Negative/neutral  Positive  Negative/neutral  Negative/neutral  Positive  Negative/neutral  Negative/neutral  Positive  Positive  Positive  Negative/neutral  Positive  Negative/neutral  Positive |
| *Aloe vera* | Dat et al, 2012  Hekmatpou et al, 2019 | 347  1409 | Negative/neutral  Positive |
| Onion extract | Hosnuter et al, 2007  Ocamp-Candiani et al, 2014  Owji et al, 2018  Willital and Simon, 2013  Beuth et al, 2006  Willital and Heine, 1994  Chung et al, 2006  Draelos et al, 2012  Prager and Gauglitz, 2018  Borgia et al, 2010  Perez et al, 2010 | 60  61  26  1268  771  45  24  44  125  30  30 | Negative/neutral  Negative/neutral  Negative/neutral  Positive  Positive  Positive  Negative/neutral  Positive  Positive  Negative/neutral  Positive |
| Green tea extract | Ud-din et al, 2021  Ud-din et al, 2019 | 62  40 | Positive  Positive |
| 5% imiquimod | Berman et al, 2005  Berman et al, 2009 | 20  20 | Negative/neutral  Negative/neutral |
| Manuka honey | Malhotra et al, 2017  Thamboo et al, 2016 | 46  21 | Negative/neutral  Negative/neutral |
| Vitamin E | Zampieri et al, 2010  Khoo et al, 2011  Baumann and Spencer, 1999  Jenkins et al, 1986 | 428  122  15  159 | Positive  Negative/neutral  Negative/neutral  Negative/neutral |
| ***Others***  Mebo scar  Bio skin oil  Vitamin D  Tranilast 8% gel  aCT1 gel  TGFb3 +HA in silicone (SKN2017B)  1,4 Diaminobutane  Topical betuline gel (TBG)  Calcium glycerophosphate (CGP)  *Hypericum perforatum*: St John’s Wort  polymixin B-bacitracin-neomycin ointment | Basson et al, 2019  Bielfeldt et al, 2018  van der Veer et al, 2009  Kohavi et al, 2017  Grek et al, 2017  Zoumalan et al, 2019  Dolynchuk and Tredget, 2020  Kindler et al, 2016  Ong et al, 2011  Samadi et al, 2010  Berger et al, 2000 | 45  80  30  26  91  45  78  220  22  144  70 | Positive  Positive  Negative/neutral  Positive  Positive  Positive  Positive  Positive  Positive  Positive  Positive |

**Table 1** Topical formulations for skin scarring. An array of topicals exist for skin scar treatment, table 1 highlights those ranking as Level 1 or Level to LOEs.
